# Mathematical Modelling and Optimization for Facile Synthesis of Structured Activated Carbon (ACs) from *Adansonia kilima* (*Baobab*) Wood Chips Integrating Microwave-Assisted Pyrolysis for the Elimination of Lead (II) Cations from Wastewater Effluents

**DOI:** 10.3390/molecules28186640

**Published:** 2023-09-15

**Authors:** Santhana Sellamuthu, Zaira Zaman Chowdhury, Khalisanni Khalid, Shahjalal Mohd. Shibly, Md Mahfujur Rahman, Masud Rana, Irfan Anjum Badruddin, H. M. T. Khaleed, Sarfaraz Kamangar, Mohd. Rafie Bin Johan, Mohamed Hussein, Ajita Mitra, Abu Nasser Faisal

**Affiliations:** 1 Nanotechnology and Catalysis Research Center, University of Malaya, Kuala Lumpur 50603, Malaysiaajitamitra@gmail.com (A.M.); faisal_dhaka2003@yahoo.com (A.N.F.); 2 Malaysian Agricultural Research & Development Institute (MARDI), Serdang 43000, Malaysia; 3 Institute of Halal Management, Islamic Business School, Universiti Utara Malaysia, Kedah 06010, Malaysia; 4 Department of Civil Engineering, World University of Bangladesh, Dhaka 1230, Bangladesh; 5 Department of Mechanical Engineering, College of Engineering, King Khalid University, P.O. Box 394, Abha 61421, Saudi Arabia; 6 Department of Mechanical Engineering, Faculty of Engineering, Islamic University, Madinah Munawwarra 42351, Saudi Arabia; 7 Department of Chiemistry, King Khalid University, P.O. Box 9004, Abha 61413, Saudi Arabia

**Keywords:** ligno-cellulosic biomass, activated carbon (AC), response surface methodology (RSM), Box–Behnken design (BBD), lead (II) cations (Pb^+2^), microwave-assisted pyrolysis (MWP), conventional pyrolysis, optimization and analysis of variance (ANOVA)

## Abstract

In this research, activated carbon (AC) was synthesized from ligno-cellulosic residues of *Adansonia kilima (Baobab)* wood chips (AKTW) using two-step semi-carbonization and subsequent pyrolysis using microwave-induced heating (MWP) in the presence of a mild activating agent of K_2_CO_3_. The influence of process input variables of microwave power (*x*_1_), residence time (*y*_1_), and amount of K_2_CO_3_ (*z*_1_) were analysed to yield superior quality carbon having maximum removal efficiencies (*R*_1_) for lead (II) cations from waste effluents, fixed carbon percentages (*R*_2_), and carbon yield percentages (*R*_3_). Analysis of variance (ANOVA) was used to develop relevant mathematical models, with an appropriate statistical assessment of errors. Level factorial response surface methodology (RSM) relying on the Box–Behnken design (BBD) was implemented for the experimental design. The surface area and porous texture of the samples were determined using Brunauer, Emmett, and Teller (BET) adsorption/desorption curves based on the N_2_ isotherm. Surface morphological structure was observed using field emission scanning electron microscopic (FESEM) analysis. Thermogravimetric analysis (TGA) was carried out to observe the thermal stability of the sample. Change in the carbon content of the samples was determined using ultimate analysis. X-ray diffraction (XRD) analysis was performed to observe the crystalline and amorphous texture of the samples. The retention of a higher proportion of fixed carbon (80.01%) ensures that the synthesized adsorbent (AKTWAC) will have a greater adsorption capacity while avoiding unwanted catalytic activity for our synthesized final sample.

## 1. Introduction

Adsorption is the most widely utilized approach for treating wastewater among all known techniques due to its ease of operation, minimal installation costs, and superior effluent treatment effectiveness [1,2,3,4]. It is a reasonably environmentally benign and sustainable strategy that utilizes waste materials, such as lignocellulosic wastes, to develop adsorbent materials. Subsequently, it leads to the reduction of environmental pollution [5,6,7]. According to the adsorption studies, activated carbon (AC) is a commonly utilized adsorbent material due to its excellent textural features; such as enlarged surface area, average pore volume, and well-defined porous structures [8,9,10].

The morphological, as well as the chemical, features of the synthesized carbon are determined by the lignocellulosic precursors [8,9], including the experimental variables under which the activation procedure has taken place [10]. Physical and chemical processes of activation, or a combination of both techniques, are used to generate activated carbons (ACs) [11,12]. The porous nature of activated carbon (AC) enables it to have a high sorption capacity. It is frequently used in industries to purify wastewater containing metallic/non-metallic impurities, colours, and organic compounds. Additionally, it has exhibited considerable performance as a catalyst support and heating substrate in thermochemical systems.

There are two processes used to produce ACs: carbonization and activation. Lignocellulosic biomass is a suitable precursor for the production of ACs. ACs can be generated using physical, chemical, and physiochemical thermochemical conversion of lignocellulosic biomass. There is one step involved in the physical and chemical process during pyrolysis. The physiochemical method involves two steps of carbonization and activation in the presence of a suitable chemical reacting agent. These processes are carried out independently, having the raw materials first carbonized and pyrolyzed at a higher temperature (400–1000 °C) in the presence or absence of physical or chemical activating agents to enhance their porosity. Nevertheless, carbonization and activation can be accomplished in a single-step process. There are mainly three types of pyrolysis process: traditional, microwave heating, and catalytic pyrolysis. Conventional fixed-bed/tubular pyrolysis with electric heating is more prevalently utilized to manufacture ACs [4]. Due to the extended period required for the pyrolysis in an inert environment, the traditional electric heating technique has economical limitations [13,14]. Additionally, due to the diameter and length of the tubular furnace, a temperature gradient exists between the upper surface of the precursors and the interior side of the sample, which may influence the composition of the activated carbons (ACs) produced [13,14].

The conventional heating method for transferring the heat may be ineffective and slow. The heat must be transmitted from the heat source (e.g., heating surface or filament) to the reactor system, and then to the thermal conductor (e.g., the gas contained within the furnace compartment) before reaching the sample surface which needs to be heated. Additionally, this leads to a considerable loss of energy, requiring additional time to heat the substances to the appropriate temperature for the pyrolysis to commence. Subsequently, microwave-assisted pyrolysis (MWP) has been described for the production of high quality ACs [15,16,17,18,19].

Microwave-induced heating provides an advantage over conventional heating in terms of faster treatment time (10–15 min approximately), which usually result in energy conservation and sustainability of the environment. This leads to a decrease in the processing expenditure, using traditional pyrolysis process [13,14]. Microwave-induced heating generates heat differently than traditional heating in a regular furnace. In a traditional heating process, heat is transferred via conduction and convection, while in MWP heating, heat is effectively produced via ion conduction and molecular dipole–dipole oscillation or rotation of the sample. Consequently, sample temperature rapidly rises and tends to produce a consistent, homogeneous temperature dispersion across the sample [13,14]. MWP heating does not involve direct interaction between the radiation supply and the feedstock. This stimulates friction/interfacial turbulence between the subatomic particles in the substance, which results in a rapid thermal energy transfer across the entire volume of the substance. This can offer additional ultra-fast, ‘volumetric’ heating to reheat the content in large quantities. Numerous researchers have used MWP to produce ACs from a variety of precursors, including durian shell [16], palm oil shell [13,20,21], banana frond [17], palm shell [18], sugarcane bagasse [15], cocoa shell [19], seed pods of *Albizia* [22,23], rambutan peel [24], peel of pomegranate [25], date stones [26], wastes from microalgae [27], stalks of lotus [28], Crofton weeds [29], endocarp of macadamia [30], sludge of pulp mill [31], industrial waste lignin [32], corn stover [33], peanut shell [34], etc.

Lead is considered as one of the hazardous heavy metals that can be ingested through exposure to water and air [35]. Even at low concentrations, it is injurious to human and aquatic organisms. It is typically found to form divalent cations, which is a highly toxic element that is widely present in surface, ground, and industrial wastewater [36]. It has the potential to be absorbed through the skin, as well as the digestive and respiratory systems, which can have an effect on the body’s physiological mechanisms. Furthermore, its deposition can lead to severe environmental and ecological threats for water bodies [37,38]. Wastewater from metal plating, paint, dye, battery production, and glass sectors can release lead cations into water [38,39]. Based on the World Health Organization (WHO), the limit of lead in water should be 0.01 mg/L. Earlier divalent cations of lead (Pb^2+^) concentration was found to be approximately 0.321 mg/L in open drainage systems in certain industrial sectors [39,40].

In this research, semi-carbonized char obtained from a fixed bed carbonization system, derived from baobab wood chips from the trunk, was activated using microwave-assisted pyrolysis (MWP), where potassium carbonate (K_2_CO_3_) was used as a chemical activating agent. The process parameters of MWP were optimized in terms of microwave power (*x*_1_), irradiation period (*y*_1_), and impregnation ratio (*z*_1_) using K_2_CO_3_. By incorporating the Box–Behnken design (BBD), removal percentages of lead, Pb (II) cations (*R*_1_), fixed carbon percentages (*R*_2_), and carbon yield percentages (*R*_3_) of each sample were measured depending on the response surface methodology (RSM). These assessments of input/independent variables are essential in determining the effectiveness of the developed method for recycling and converting the waste chips using a more energy-efficient process to yield high-quality carbon that can be used for the removal of Pb (II) cations from polluted streams. In previous literature, alkaline hydroxide, ZnCl_2_, or H_3_PO_4_ acid, which are strong Lewis acid-based activating agents, have been used for pyrolysis using MWP as well as the conventional heating process. Furthermore, ZnCl_2_ itself can introduce secondary pollutants in aqueous effluents after leaching out from the carbon substrate. *Adansonia kilima* is mainly found in southern and eastern Africa. Later on, it was also found in Kenya, Tanzania, Zambia, South Africa, and Namibia. Previously, *Adansonia digitata*, another kind of Baobab stem powder, and shell were activated using highly concentrated ZnCl_2_ to produce ACs using conventional heating [41]. *Adansonia digitata* seed hulls, fruit shells, and fibres were also activated using conventional heating using KOH, ZnCl_2_, H_3_PO_4_ activation; steam pyrolysis; and two-step conventional pyrolysis, whereby both stages were conducted at a very high temperature of 800–900 °C, respectively [42,43,44]. The use of harsh, corrosive chemicals (KOH, ZnCl_2_, H_3_PO_4_) in the production of ACs is not economically feasible. Nevertheless, the conventional pyrolysis technique is time consuming. In this study, the potential of a relatively mild activating agent (K_2_CO_3_) is explored for microwave-assisted pyrolysis (MWP) to produce ACs with outstanding physiochemical properties. To the best of our knowledge, no research has been reported regarding the application of the suggested process of microwave-assisted pyrolysis (MWP) using a Box–Behnken design (BBD) for producing ACs from *Adansonia kilima* (*Baobab*) wood chips (AKTW) using the mild activating agent of K_2_CO_3_.

## 2. Results

### 2.1. Regression Model Development and Statistical Analysis

Table 1 and Table 2 illustrate the magnitude of operational parameters during the microwave-assisted pyrolysis (MWP) process. The design matrix based on a Box–Behnken design (BBD) design suggested 17 experimental runs under different conditions containing three input variables (power—*x*_1_, residence time—*y*_1_, and ratio—*z*_1_) and three output variables/responses (removal percentages—*R*_1_, fixed carbon content—*R*_2_, and yield percentages—*R*_3_). Five experimental runs under identical conditions were performed for the interpretation of the standard error at the centre/zero position.

Three quadratic regression models based on removal percentages—*R*_1_, fixed carbon content—*R*_2_, and percentage yield—*R*_3_ are suggested by the software and represented by Equations (1)–(3).

(1) $$Removal\;Percentages\;of\;Lead,\;Pb\left(II\right)Cations\;(R_{1})=+85.86-4.06x_{1}-3.83y_{1}-4.25z_{1}-1.51x_{1}y_{1}\;-2.74x_{1}z_{1}+2.73y_{1}z_{1}-1.21x_{1}^{2}-1.62y_{1}^{2}-1.62z_{1}^{2}$$

(2) $$\begin{array}{cc} Fixed\;Carbon & Percentages\;\left(R_{2}\right)\\ & =+79.06-5.68x_{1}-2.22y_{1}-1.60\;z_{1}+2.70x_{1}y_{1}+1.11\;x_{1}z_{1}+1.54y_{1}z_{1}-7.44x_{1}^{2}\\ & -3.31y_{1}^{2}-3.90z_{1}^{2} \end{array}$$

(3) $$\begin{array}{cc} Carbon\;Yield & Percentages\;\left(R_{3}\right)\\ & =+83.57-3.30\;x_{1}-0.95y_{1}-2.65z_{1}+0.17\;x_{1}y_{1}-0.77\;x_{1}z_{1}-0.32\;y_{1}z_{1}\\ & -1.05x_{1}^{2}-0.31\;y_{1}^{2}-2.48\;z_{1}^{2} \end{array}$$

**Figure 1:**
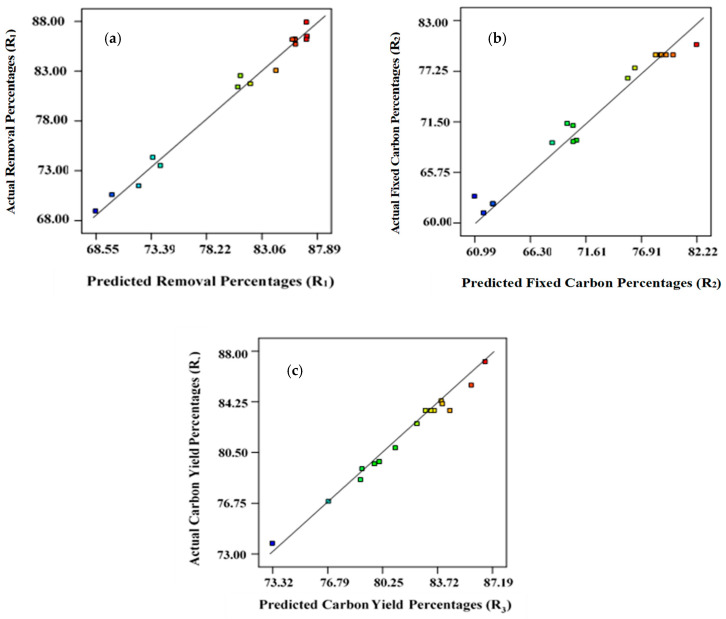
Predicted versus actual/experimental data points: (**a**) removal percentages, *R*_1_; (**b**) fixed carbon percentages, *R*_2_; (**c**) yield percentages, *R*_3_.

The R^2^ values for Equations (1), (2), and (3) are 0.987, 0.981, and 0.972, respectively, which were closer to unity, indicating that the developed models are suitable (Figure 1). The coefficient of variation (CV) and standard deviations found were minimal, indicating that the proposed models are reliable (Table 3). The signal-to-noise ratio was used to estimate the magnitude of adequate precision [45,46]. Adequate precision values need to be higher than 4 for successful simulation of the proposed models. For removal percentages, *R*_1_, fixed carbon content, *R*_2_, and yield, *R*_3_, the adequate precision was 21.76, 18.96, and 20.26, respectively. Therefore, the experimental data set reported here is statistically significant for navigating the design [47].

Table 4, Table 5 and Table 6 present the findings from ANOVA analysis for removal percentage (*R*_1_), carbon content percentages (*R*_2_), and carbon yield percentages (*R*_3_), correspondingly, indicating that these regression models are statistically relevant, having a confidence level at 95%. To assess the level of competence of the developed models, the magnitude of F-test results, reflecting the divergence of the data from the average/mean value, were computed. Figure 1 represents linear plots for predicted versus actual data points for *R*_1_, *R*_2_*,* and *R*_3_, respectively.

The model F values for Pb (II) cations removal percentages (*R*_1_), fixed carbon content percentages (*R*_2_), and yield percentages (*R*_3_) were 60.45, 56.64, and 31.34, respectively, indicating that these models were credible and significant. Nevertheless, for these output responses, the magnitude of Prob > F values were lower than 0.05, indicating that the model variables included here for the analysis of responses were significant [48].

The linear variables of power (*x*_1_), time (*y*_1_) and ratio (*z*_1_), interaction terms *x*_1_*y*_1_ and *y*_1_*z*_1_, and also their quadratic terms *y*_1_^2^ and *z*_1_^2^, were significant for the response of removal percentages, *R*_1_. In comparison to power (*x*_1_), other linear factors such as time (*y*_1_) and ratio (*z*_1_) had a moderate effect on the elimination percentages (*R*_1_). The interaction between power and ratio (*x*_1_*z*_1_) had a stronger influence on removal percentages, *R*_1_ (according to the greatest value of F = 30.09) than the remaining interaction variables of *y*_1_*z*_1_ and *z*_1 *×* 1_.

For fixed carbon content percentages, *R*_2_, power (*x*_1_), time (*y*_1_), and ratio (*z*_1_), along with the interaction terms of *x*_1_*y*_1_ and *y*_1_*z*_1_, are significant model terms. The quadratic model terms *x*_1_^2^, *y*_1_^2^, and *z*_1_^2^ were also significant (Table 5). The power (*x*_1_), radiation duration (*y*_1_), and ratio (*z*_1_*)* had a substantial impact on percentage yield of carbon (*R*_3_). The quadratic terms of *x*_1_^2^ and *z*_1_^2^ were also significant for *R*_3_. The interaction between time and ratio, *y*_1_*z*_1_, had a more prominent influence compared with the other interaction terms (*x*_1_*z*_1_ and *x*_1_*y*_1_) on percentage yield, *R*_3_ (Table 6).

### 2.2. Process Optimization

**Figure 2:**
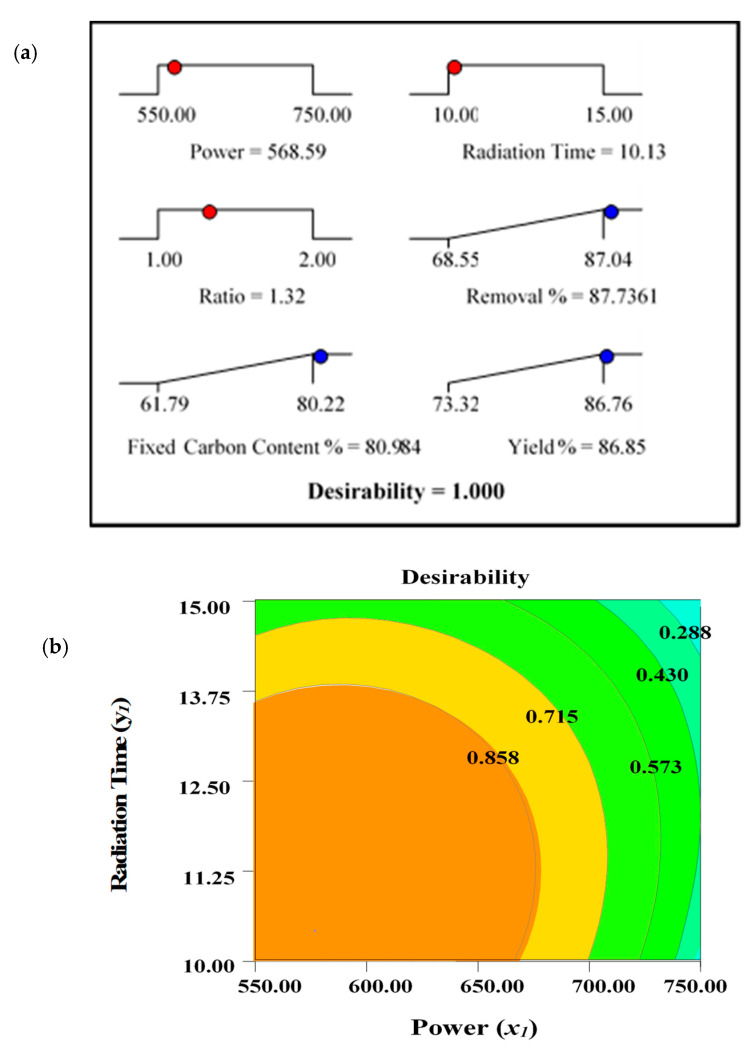
(**a**) Optimization/desirability ramp and (**b**) 2D contour plots for desirability function.

The optimal values for the output variables, designated as responses (removal percentages, *R*_1_, fixed carbon content percentages, *R*_2_, and yield percentages, *R*_3_), were determined. Due to the distinct interest areas of input variables (power—*x*_1_, radiation time—*y*_1_, and ratio—*z*_1_), it was difficult to maximize the selected responses (*R*_1_, *R*_2_, and *R*_3_) using the identical operating conditions. Maximum removal percentages, *R*_1_, and fixed carbon percentages, *R*_2_, were observed under identical conditions shown at Run 6 (power, 550 watts, time, 10 min, and ratio 1.50). This is expected, as the maximum amount of fixed carbon content with less ash residues can ensure maximum removal percentages. However, yield percentage, *R*_3_, was maximum under the condition of Run 11 (power, 550 watts, time, 12.5 min, and ratio, 1.00). Two different sets of parameters were observed for maximum values of *R*_1_, *R*_2_, and *R*_3_. Thus, the desirability indicator was implemented using the State Ease Design Expert Software–9. To determine the optimal set of processing variables for the production of ACs, the target criterion for the output variables/responses were fixed to “maximum”, while the level of input variables were kept “in the range”. The desirability ramp with the graph is shown in Figure 2.

The predicted and actual experimental values for the responses determined under optimum processing conditions are summarised in Table 7. The measured values demonstrate the average of the three experimental results carried out for the assessment of the accuracy of the process.

### 2.3. Effect of Process Variables on Adsorption Performance (R_1_) of AKTWAC

The optimum ACs need to ensure higher removal efficiencies of contaminants from the aqueous phase, having superior physicochemical properties. In such scenarios, the carbon materials (AKTWAC) ought to have significant BET surface area and suitable porosity to enable the aqueous state sorption process. RSM was used to explore the influence of three factors (power—*x*_1_, residence time—*y*_1_, and ratio—*z*_1_) on the removal percentages of Pb (II) metal ions (*R*_1_), employing a three-dimensional response surface (RSM) mesh and two-dimensional contour plots. According to the RSM analysis, all three factors contributed successively to increasing the percentage removal (*R*_1_) up to a specified level.

**Figure 3:**
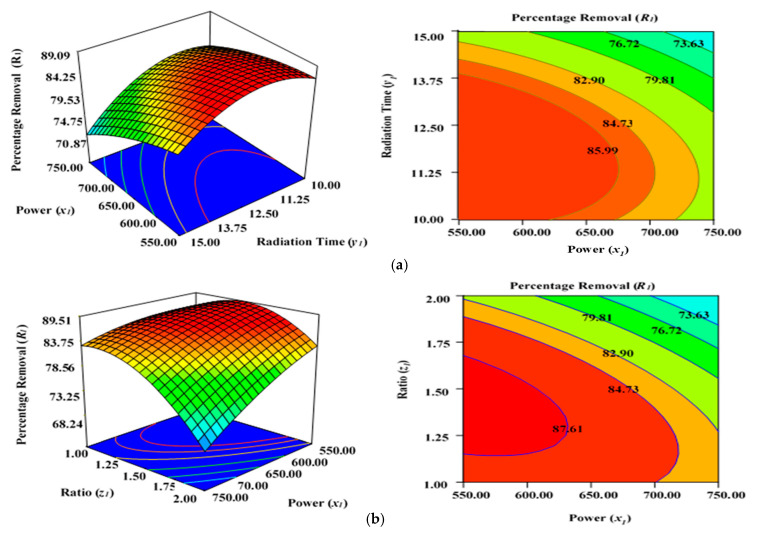

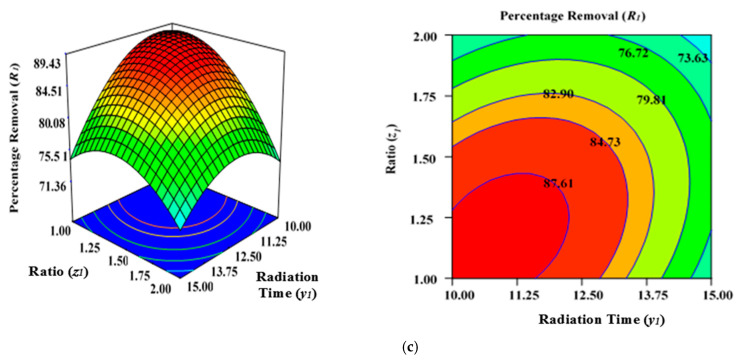
Three-dimensional RSM surface mesh plots with respective 2D contour plots for removal percentages (*R*_1_): (**a**) effect of microwave power (*x*_1_) and radiation duration (*y*_1_) when impregnation ration (*z*_1_) was kept consistent at the centre/zero level (1.5), (**b**) effect of duration (*y*_1_) and ratio (*z*_1_) when power (*x*_1_) was fixed at the centre point (650 watts), (**c**) effect of power (*x*_1_) and ratio (*z*_1_) when radiation time (*y*_1_) was kept constant at the centre level (12.5 min).

Figure 3a illustrates the effect of microwave power (*x*_1_) and radiation duration (*y*_1_) on the percentage removal (*R*_1_) when the other parameters of impregnation ration (*z*_1_) were kept consistent at centre/zero level (1.5). Figure 3b depicts the impact of duration (*y*_1_) and ratio (*z*_1_) on the percentage removal (*R*_1_) when power (*x*_1_) was fixed at the centre point (650 watts). Figure 3c shows the cumulative impact of power (*x*_1_) and ratio (*z*_1_) on the removal percentages (*R*_1_) when radiation time (*y*_1_) was kept constant at the centre level (12.5 min). The values provided along the *x-* and *y*-axis are precise, actual values.

Figure 3a–c demonstrates that increasing the radiation power (*x*_1_) as well as extending the retention period (*y*_1_) in the microwave improves the percentage removal (*R*_1_) up to a definite point. This inevitably results in a concave layout of the RSM figures. The greatest percentage removal (*R*_1_) achieved was approximately 87.04% (from experimental Run 6; condition: lower power level (*x*_1_)—550 watts, duration(*_y_*_1_)—10 min, and ratio (*z*_1_)—1.5 under N_2_ flow of 50 mL/min). The application of microwave power (*x*_1_) for a certain duration (*y*_1_) and ratio (*z*_1_) can increase the elimination and dehydration processes during the MWP pyrolysis/activation phase. Enhanced power (*x*_1_) and duration (*y*_1_) of activation can ensure the emission of more volatile chemicals from the interior side of the carbon matrix. To increase the porosity and surface area, an optimal contact period is necessary to allow greater interaction between the semi-carbonized char (AKTWC) and K_2_CO_3_ in the presence of N_2_ gas [49,50,51]. On the other hand, a too low ratio (*z*_1_) cannot enhance the cracking mechanism necessary for the formation of pores, consequently having no favourable leverage on the sorption performance of the ACs synthesised here.

The lower removal percentages (*R*_1_) at a very high level of MWP power (*x*_1_) could be related to the sintering/thermal decomposition, accompanied by deformation of the semi-carbonized char (AKTWC). This can cause realignment of the synthesised activated carbon structure, leading to reduced surface areas and pore volume. This phenomenon was explained earlier for the sorption of MB using ACs produced from the stalks of cotton using MWP-based KOH activation, whereby 400-watt power was chosen as the optimal level of power for activation [52]. The findings of ANOVA results in Table 3 reveal that ratio (*z*_1_) has the major impact on Pb (II) cation uptake [49,50].

The cumulative effect of power (*x*_1_) and ratio (*z*_1_) is decreasing the removal percentages (*R*_1_), as can be seen by the contours of the graphs (Figure 3b), until a certain range. This indicates that increasing the power (*x*_1_) and ratio (*z*_1_) of activating salt in the environment of N_2_ gas would improve the rate of the reaction between K_2_CO_3_ and semi-carbonized char (AKTWC), leading to more porosity, up to a fixed threshold value. Maximizing the power (*x*_1_) and ratio (*z*_1_) beyond that specific threshold point will have a detrimental impact on the sorption process. This has been anticipated because a very high level of power or ratio of K_2_CO_3_ can disrupt certain functional groups over the carbon surface. Microwave annealing due to heat may negatively affect the porous structure of the synthesized carbon, incinerating the pore walls and decreasing the overall volume of the pore [44,53,54].

The residence time (*y*_2_) also had a progressive effect on increasing the surface area, with a porous texture of the ACs produced from cassava peel. Meanwhile, the proportion of micro- and mesopores was significantly influenced by the impregnation ratio using the strong base of KOH and temperature [49,50,51]. Enhanced radiation time (*y*_1_) and power (*x*_1_) decreased the removal percentages (*R*_1_) after a certain level. At a higher power level (x_1_) of 650 to 750 watts and residence time (y_1_) of approximately 12.5 to 15 min, more radiation takes place, and the semi-carbonized char (AKTWC) is exposed under that condition. This may disrupt the C–C and C–O–C bonds of carbon and can cause burning of the carbon to produce more ash and weight loss. Too much exposure under microwave radiation is not recommended because it may increase ash production, destruction of pores, and functional groups, resulting in a reduction in the removal efficiencies (*R*_1_).

Thereby, it can be inferred that exceeding the optimal limits for radiation power (*x*_1_), duration (*y*_1_), and ratio (*z*_1_) destroys the porous network, which tends to result in a decreased BET surface area and reduced removal rate percentages (*R*_1_). Nevertheless, the removal percentages (*R*_1_) were reduced at extremely minimal levels of ratio (*z*_1_) of approximately 1 at a power (*x*_1_*)* of 650 or 750 watts, irrespective of duration (*y*_1_) at 10 to 15 min (Table 2). This occurs as a result of inadequate interactions between the semi-carbonized char (AKTWC) and K_2_CO_3_, potentially degrading the efficiency of the synthesized carbon (AKTWAC) [55].

### 2.4. Effect of Process Variables on Fixed Carbon Content (R_2_)

**Figure 4:**
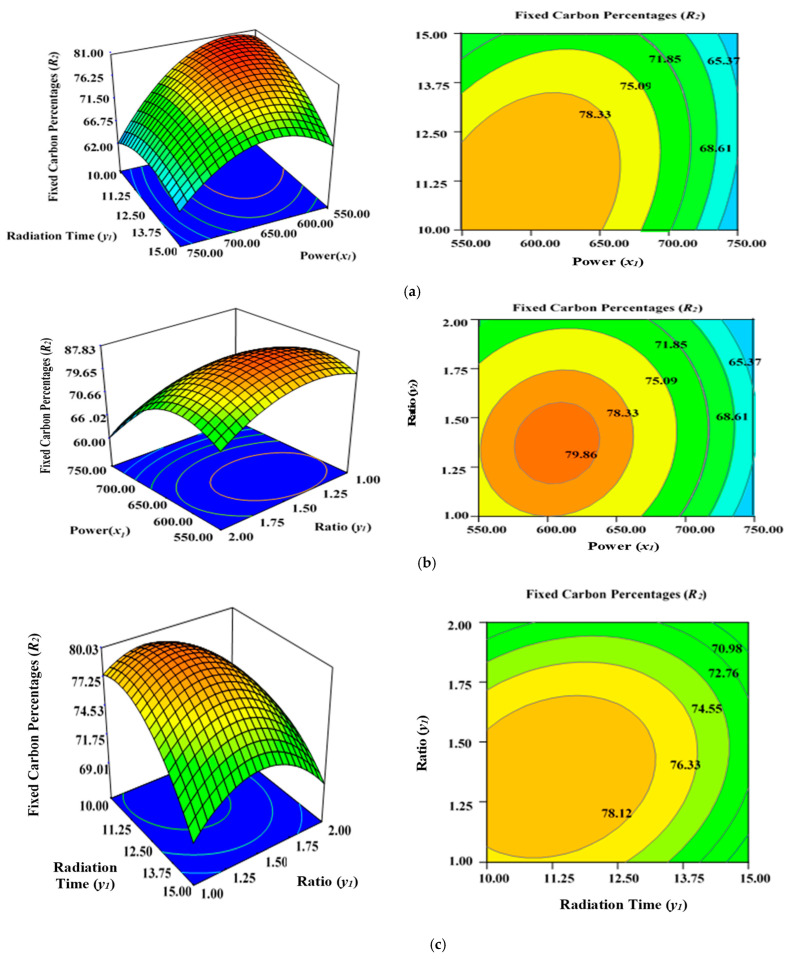
Three-dimensional RSM surface mesh plots with respective 2D contour plots for fixed carbon percentages (*R*_2_): (**a**) effect of microwave power (*x*_1_) and radiation duration (*y*_1_) when impregnation ration (*z*_1_) was kept consistent at the centre/zero level (1.5), (**b**) effect of duration (*y*_1_) and ratio (*z*_1_) when power (*x*_1_) was fixed at the centre point (650 watts), (**c**) effect of power (*x*_1_) and ratio (*z*_1_) when radiation time (*y*_1_) was kept constant at the centre level (12.5 min).

Figure 4a illustrates the incremental effect of two variables, power (*x*_1_) and radiation time (*y*_1_), on the percentage fixed carbon (*R*_2_) inside the AC samples produced using the condition of experimental Runs 1–17 (Table 2), whereby K_2_CO_3_ ratio (*z*_1_) was set to zero at the centre point (1.25). Figure 4b depicts the simultaneous effect of radiation time (*y*_1_) and K_2_CO_3_ ratio (*z*_1_) on percentage fixed carbon (*R*_2_), with power (x_1_) maintained at its respective centre point (650 watts). Figure 4c shows the collective effect of power (*x*_1_) and K_2_CO_3_ ratio (*z*_1_) on percentage fixed carbon (*R*_2_) when time (*y*_1_) was maintained at its respective centre point (12.50 min).

In this study, all three factors examined had a stimulating effect on the fixed carbon content percentages (*R*_2_). It was discovered that increasing the power (*x*_1_), duration (*y*_1_), and ratio (*z*_1_) significantly lowered the carbon content (*R*_2_) of the sample after the optimal limit. Power (*x*_1_) had the greatest effect on fixed carbon content (*R*_2_), as demonstrated before by the maximum F value of 258.10 in Table 4, but ratio (*z*_1_) was relatively less significant (F value is 20.38) in comparison to power (*x*_1_) and time (*y*_1_). The lowest carbon content was observed at 750 watts for 15 min, with a K_2_CO_3_ ratio of 1.50 (Run 15), as indicated by Table 2. Increasing the power (*x*_1_), radiation duration (*y*_1_), and ratio (*z*_1_) after a certain level will accelerate the diffusion K_2_CO_3_ within the char matrix, leading to the formation of ash. This would lower the fixed carbon percentages (*R*_2_) due to enhanced de-volatilization and burning of the carbon to ash residues. This phenomenon is consistent with previous findings when producing ACs from orange peel using microwave-induced KOH and NaOH activation [56].

### 2.5. Effect of Process Variables on Yield Percentages of AKTWAC Carbon (R_3_)

**Figure 5:**
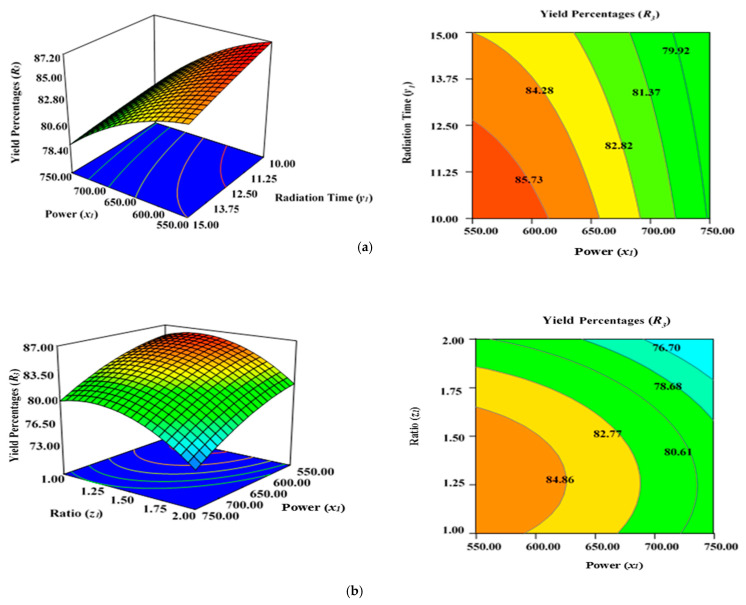

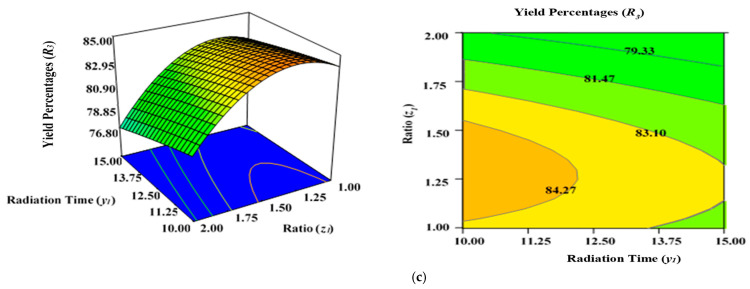
Three-dimensional RSM surface mesh plots with respective 2D contour plots for yield percentages (*R*_3_): (**a**) effect of microwave power (*x*_1_) and radiation duration (*y*_1_) when impregnation ration (*z*_1_) was kept consistent at the centre/zero level (1.5), (**b**) effect of power (*x*_1_) and ratio (*z*_1_) when radiation time (*y*_1_) was kept constant at the centre level (12.5 min), (**c**) effect of duration (*y*_1_) and ratio (*z*_1_) when power (*x*_1_) was fixed at the centre point (650 watts).

Figure 5a demonstrates the combined effect of two variables, power (*x*_1_) and radiation time (*y*_1_), on the percentage yield of carbon (*R*_3_) inside the AC samples produced using the condition of experimental Runs 1–17 (Table 2), whereby the K_2_CO_3_ ratio (*z*_1_) was fixed to the centre at 1.25. Figure 5b represents the concurrent effect of radiation time (*y*_1_) and K_2_CO_3_ ratio (*z*_1_) on percentage carbon yield (*R*_2_), with power (*x*_1_) kept at its respective centre point (650 watts). Figure 5c shows the collective effect of power (*x*_1_) and K_2_CO_3_ ratio (*z*_1_) on percentage carbon yield (*R*_3_) when time (*y*_1_) was retained at its respective centre point (12.50 min).

All three variables considered in this study had a significant impact on the carbon yield percentages (*R*_3_). It was observed that raising the power (*x*_1_), time (*y*_1_), and ratio (*z*_1_) of the MWP pyrolysis of the sample resulted in a substantial decrease in the yield percentages (*R*_3_) after the optimum limit. As previously indicated by the maximum F value of 87.38 in Table 5, power (*x*_1_) had the highest impact on carbon yield (*R*_3_), while radiation time (*y*_1_) was considerably less significant (F value of 7.20). Ratio (*z*_1_) had a stronger effect on yield percentages (*R*_3_) compared with time (*y*_1_). As stated in Table 2, the lowest carbon yield was found at 750 watts for 12.5 min with a K_2_CO_3_ ratio of 2.00 (Run 15).

A higher magnitude of power (*x*_1_) in conjunction with a greater ratio (*y*_1_) results in lower carbon yield percentages (*R*_3_) (Figure 5a). The rise in power (*x*_1_) will induce more heat energy and will eventually intensify the elimination and dehydration activities inside the sample, resulting in the formation of more gaseous and liquid fractions rather than solid ACs with a higher proportion of ash residues. Thus, yield percentage will drop at higher power levels with enhanced radiation time. This observation was previously reported in the literature when oil palm fronds were used to prepare ACs using the conventional heating approach for the elimination of zinc (Zn (II)) cations [57]. Overall, the yield percentages (*R*_3_) of ACs dropped when the level of the process parameters was increased. Several studies have documented a similar tendency [58,59,60]. The negative relationship between microwave radiation duration and AC production can be explained by the increasing amount of breakdown of organic residues by prolonged radiation time (heating) [60]. As a result, more volatile content was discharged from the partially carbonized char, resulting in a reduced yield of ACs. The greater proportion of K_2_CO_3_, along with the increased thermal energy generated by the higher microwave power, likely aided the burning of the semi-carbonized char, resulting in a reduced conversion of char to AC substrate.

### 2.6. Physio-Chemical Characterizations

#### 2.6.1. Surface Morphological Analysis

**Figure 6:**
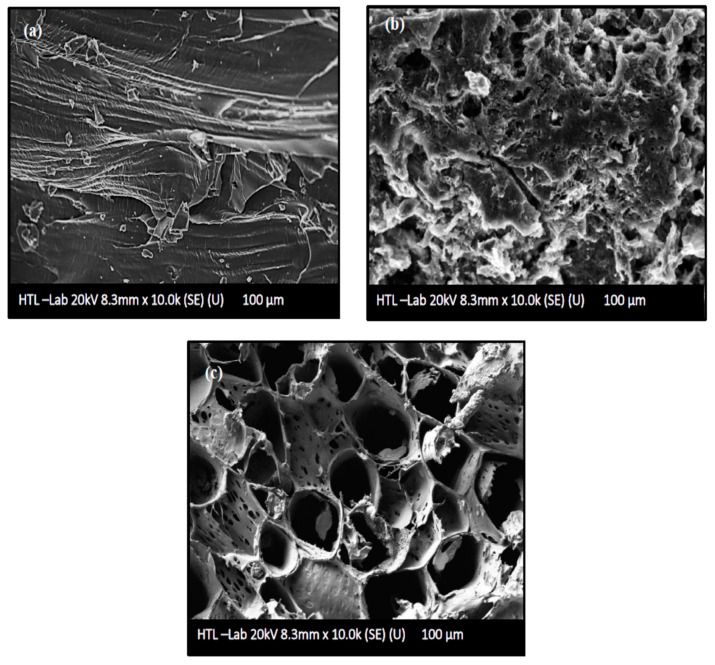
Surface morphological analysis using field emission scanning electron microscopic (FESEM) images: (**a**) AKTW, (**b**) AKTWC, (**c**) AKTWAC.

FESEM images of untreated AKTW, AKTWC, and AKTWAC produced under optimal circumstances are shown in Figure 6a–c. As can be seen from Figure 6a, the untreated precursor of AKTW has a relatively smooth surface with few cracks and craters and a negligible amount of pores. It is almost non-porous before the semi-carbonization process using the fixed bed reactor. The semi-carbonization process in the presence of N_2_ gas at 650 °C has created some pores over the surface of AKTWC (Figure 6b). Microwave-assisted pyrolysis (MWP) in the presence of K_2_CO_3_ leads to the formation of many distorted pores. However, the majority of these pores have a circular shape (Figure 6c)

The gasification process in the presence of K_2_CO_3_ is responsible for the development of the porous texture of the carbon and can be represented by the following reactions [61]. Due to the reducing effect, the carbon can generate K_2_O, K, CO, and CO_2_ gas from K_2_CO_3_ based on the following reactions [61]:

K_2_CO_3_ + 2C → 2K + 3CO

K_2_CO_3_ → K_2_O + CO_2_

K_2_O + C → 2K + CO

It is expected that metallic potassium (K) produced during the gasification will penetrate into the internal surface of the char cavity, enlarging the existing holes, which can form new pores. Pore diameter can be regulated by specific surface interactions and the quantity and rate of volatile compounds emitted. Evidently, the volume and geometry of the pores are highly dependent on, not only the magnitude of radiation time, ratio, reactor design, flow rate, and types of the activating gas, but also on the sort (conventional or microwave-assisted) and rate of heating. After microwave-assisted pyrolysis (MWP), the microstructure of pores was clearly apparent (Figure 6c). This indicates that K_2_CO_3_ impregnation and soaking had annihilated the undesirable particles that had been clogging the pores after the semi-carbonization process (Figure 6b) [56,57]. As a result, the surface area and volume of the pores were significantly enhanced after the microwave-assisted pyrolysis process. This phenomenon is further supported by BET analysis of the AKTWC and AKTWAC sample. The final sample of AKTWAC was washed several times to ensure the removal of unreacted K_2_CO_3_ from sample. As depicted earlier, metallic potassium intercalated during microwave-assisted pyrolysis to enlarge the surface area according to mechanisms (I), (II), and (III). EDX analysis was carried out, and after final washing in AKTWAC, the potassium content was relatively small, approximately 1.02%, with carbon at 83.23%, oxygen at 5.57%, silicon at 6.34%, aluminium at 3.54%, and a trace amount of iron content at 0.30%. Other elements, such as silicon, aluminium, and iron, were found due to the presence of ash, as impurities inside the carbon matrix [35]. The lower content of potassium is due to the vigorous washing of the final sample (AKTWAC) until the pH became neutral.

#### 2.6.2. Surface Area and Porous Texture Analysis

**Figure 7:**
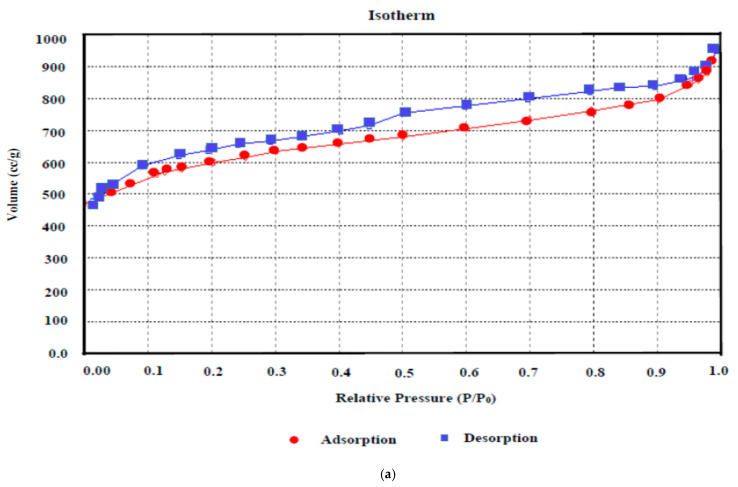

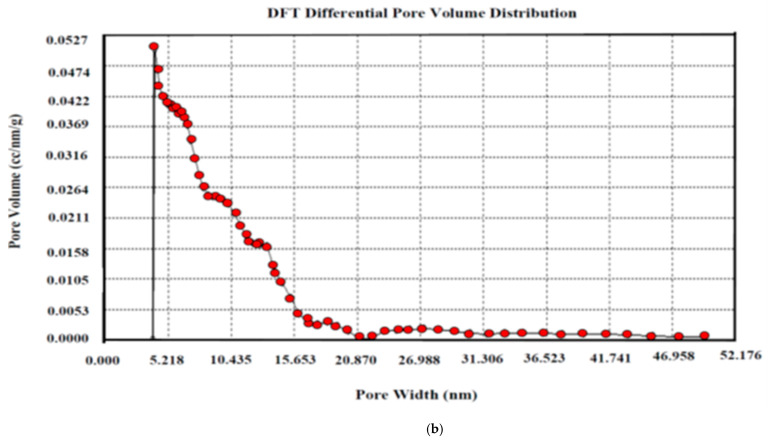
(**a**) BET adsorption isotherm and (**b**) pore size distribution of AKTWAC sample.

N_2_ adsorption–desorption curves reveal qualitative feedback on the adsorption capacity and porosity of the carbonaceous substrate. The N_2_ adsorption isotherm assessment (Figure 7a) revealed that the isotherm obtained here for AKTWAC can be classified as an intermediate between Category I and Category II isotherms, designated by the International Union of Pure and Applied Chemistry (IUPAC) association. This adsorption pattern indicates a hybrid structure containing both micro- and meso-porous texture. At relatively higher pressures, the isotherm curve exhibits a small hysteresis loop, indicating a meso-porous texture.

Table 8 provides detailed information regarding the surface area and porous texture of the AKTWC and AKTWAC samples. Mesopores have encompassed approximately 49.06% of the total volume of the pores in AKTWAC. This reflects an adequate porous texture suitable for aqueous phase adsorption.

When AKTWAC was compared to carbonized AKTWC, it was observed that the BET surface area, micropore surface area, Langmuir surface area, and total pore volume all increased significantly, confirming pore formation and enlargement of the existing ones during the microwave pyrolysis step (Table 8). The AKTWAC produced in this work had a relatively higher BET surface area of 1390.76 m^2^/g and total pore volume of 0.9643 cm^3^/g, which is efficient enough for the removal of Pb (II) cations from waste water.

**Figure 8:**
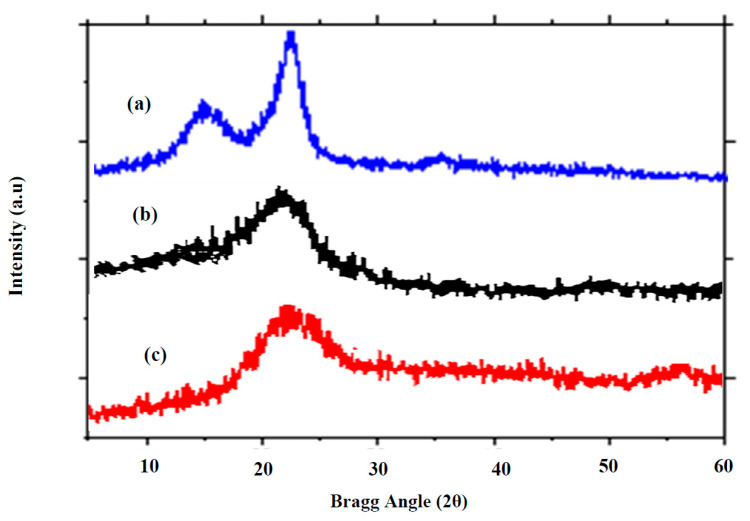
X-ray Diffraction Spectra for (**a**) AKTW (**b**) AKTWC (**c**) AKTWAC.

The pore size distribution is a representation of the intricate hollow areas inside the interior surface of the solid substrate. The pores are classified as micropores (diameter less than 2 nm), mesopores (diameter equal to 2–50 nm), and macropores (diameter greater than 50 nm) according to the IUPAC classification. The pore size distribution of AKTWAC was determined using density functional theory (DFT) modelling. The highest peak with adsorbed volume was observed between 5 and 7 nm, with an average diameter of the pore of approximately 6.73 nm (Figure 8b), indicating that the largest proportion of pores developed inside the AKTWAC were mesopores.

#### 2.6.3. Thermogravimetric and Elemental Analysis

Thermo-gravimetric (TGA) analysis was used to determine the thermal stability of AKTW, AKTWC, and AKTWAC. AKTWAC showed increased thermal stability compared with AKTWC and AKTW. As shown in Table 9, the *dtg_max_* values for AKTW, AKTWC, and AKTWAC were 347.38 °C, 371.22 °C, and 378.87 °C, respectively. Between the three primary components of ligno-cellulosic wastes, hemicelluloses had the minimum thermal stability [62,63,64]. Semi-carbonization as well as microwave-assisted pyrolysis of AKTW eliminated hemicelluloses and the amorphous portion of the cellulose. This finally increased the thermal stability of the AKTWAC sample. Thus, AKTWAC contained a greater portion of stabilized carbon fragments and was more resistant to heat. The first degrading phase, which tends to occur between 70 and 130 °C, is due to the moisture evaporation. The second degradation phase for AKTW occurs between 200 °C and 300 °C, which reflects the degradation of hemicellulose. At 300 to 400 °C, degradation of cellulose takes place. It was previously established that hemicellulose degradation occurs concurrently with degradation of cellulose in ligno-cellulosic residues. However, degradation of lignin takes place between 200 and 800 °C [62,63,64].

This effect is also noticeable from ultimate analysis, wherein the percentage carbon in AKTW was originally 50.87% but was increased to 81.99% after the consecutive process of semi-carbonization and microwave-assisted activation (MWP). Elemental analysis was carried out and is tabulated in Table 9. The CHNOS analysis revealed that successive treatment with pyrolysis increased the carbon content of the sample, but the proportion of hydrogen and oxygen decreased. Enhancement of carbon content in activated AKTWAC illustrated that microwave-assisted pyrolysis after K_2_CO_3_ impregnation was appropriate enough to enhance the quality of the final activated carbon required for sufficient removal of lead cations from water. Pyrolysis under microwave caused vigorous reactions to take place due to metallic potassium intercalation inside the carbon matrix, releasing a significant amount of volatile organic compounds as gaseous and liquid products. This finally reduced the amount of hydrogen and oxygen content inside the sample [51,64].

#### 2.6.4. X-ray Diffraction Analysis

The technique of X-ray diffraction analysis (XRD) is used in materials research to identify the crystalline properties of a material [65,66,67]. The structural variations of the AKTW, AKTWC, and AKTWAC were investigated using XRD analysis. The XRD patterns for the samples are shown in Figure 8. Owing to the stacking arrangement of the aromatic layers, a blunt diffraction peak appeared at 2θ = 22–25° for AKTWC and AKTWAC, corresponding to the diffraction of (002). This represents the amorphous texture of the AKTWC and AKTWAC samples. However, the AKTW sample contains two diffraction peaks at 16° and 22°, reflecting the presence of a crystalline and amorphous region of cellulose. After semi-carbonization and microwave-assisted pyrolysis, the microcrystalline framework of the cellulose in untreated AKTW has been disrupted. A similar trend was observed for ACs obtained from lotus stalk after the activation process using phosphorous oxy-acids [68].

## 3. Materials and Methods

### 3.1. Materials

#### 3.1.1. Preparation of Feedstock

AKTW trunk chips were obtained from a local furniture factory in Africa. The chips were washed under running water to avoid contamination by dust and dried for 24 h at 120 °C. The dried chips were crushed and ground to powder and stored in plastic vials prior to semi-carbonization using a fixed-bed reactor under nitrogen (N_2_) flow at a temperature of 650 °C for 2 h. The temperature ramping was kept constant at 10 °C per minute to avoid excessive burn off to have a lower amount of ash residues and prevent the weight loss of activated carbon (ACs). The semi-carbonized char designated as AKTWC was impregnated with K_2_CO_3_ at different ratios based on the BBD design matrix, as illustrated by Table 1 and Table 2. With that impregnated sample, 500 mL of distilled water was added and stirred at room temperature for 8 h. The mixture was kept for 24 h after the stirring to ensure adequate soaking of the sample. It was filtered and dried in a vacuum oven overnight before the microwave-assisted pyrolysis (MWP). An impregnated and soaked sample (AKTWC) was placed inside the quartz reactor of the microwave equipped with a thermocouple by changing the power (*x*_1_), residence time (*y*_1_), and ratio (*z*_1_), as suggested by Table 2. Inside the microwave, N_2_ gas flow was maintained at 50 mL/min. Each experimental run carried out under different conditions yielded different types of activated carbon (AC) samples. A synthesized AKTWAC sample after the microwave-assisted heating process was washed vigorously with distilled water to remove the residual salt. Washing was carried out until the sample pH was neutral. The final sample (AKTWAC) was dried overnight in a vacuum oven at 45 °C and sent for necessary characterizations. Lead (II) cations, Pb (II) removal percentages (*R*_1_), fixed carbon content (*R*_2_), and yield percentages of carbon (*R*_3_) were determined and are tabulated in Table 2. For further mathematical modelling, ANOVA analysis and process optimization were performed on the final sample of AKTWAC.

#### 3.1.2. Preparation of Adsorbate Solution

Lead nitrate, Pb(NO_3_)_2_, salt, and K_2_CO_3_ were purchased from Sigma–Aldrich, Japan. A stock solution of lead (II) cations with a concentration of 1000 ppm was prepared using distilled water (DI). The stock solution was diluted to 100 ppm before observing the performance of ACs prepared using two-step microwave-assisted (MWP) pyrolysis of semi-carbonized char (AKTWC).

### 3.2. Methodology

#### 3.2.1. Adsorption Studies

The concentration of 100 mg/L was agitated with 0.25 g of the AKTWAC sample to calculate the removal percentages, *R*_1_. (Table 2). At 30 °C and 200 rpm rotation speed, the adsorption experiment was carried out. The pH of the pollutant Pb (II) solution was fixed to 5.5 before agitation, and volume treated with 0.25 g of AKTWAC containing Pb (II) cations with a concentration of 100 mg/L was 50 mL until equilibrium time (6 h—after which carbon became exhausted and no further adsorption took place). The following equation [51,64,69,70] was used to determine the quantity of Pb (II) ions adsorbed onto the solid surface of ATWAC:

(4) $$q_{e\;}=\frac{(C_{0}-C_{e})\;V}{W}$$

where he quantity of cation loaded after the equilibration time is denoted by *q_e_* (mg/gm); *C_0_* represents the initial concentration Pb (II) cation; *C_e_* (mg/L) is the remaining liquid phase concentrations present after equilibrium is achieved; *V* (L) is the pollutant volume; and *W* (gm) denotes the weight of AKTWAC used. Based on Table 2, the subsequent equation was used to calculate removal percentages under various experimental conditions [51,64,69,70]:

(5) $$Removal\;Percentages=\frac{\left(C_{0}-C_{e}\right)}{C_{0}}\times 100\;$$

#### 3.2.2. Design of Experiment Using Response Surface Methodology (RSM)

To investigate the influence of several effective variables on Pb (II) cation removal efficiency (*R*_1_) from aqueous solution, the Box–Behnken design (BBD) approach, which relies on the RSM technique, was used. The BBD design is suitable for identifying the most optimal permutations of factors; at the same time, it can ensure maximum fixed carbon content (*R*_2_) with maximum yield (*R*_3_) while maintaining the highest adsorption capacity (*R*_1_). It is employed to generate a second order polynomial model including linear, interaction, and quadratic terms (Equation (6)) [66]. The independent/input variables used here for microwave-assisted pyrolysis (MWP) were power (*x*_1_), time (*y*_1_), and ratio (*z*_1_). The percentage removal of Pb (II) ions (*R*_1_), fixed carbon content (*R*_2_), and yield (*R*_3_) were selected as responses (dependent variable). Fixed carbon content was measured using TGA analysis.

To conclude, the optimization technique consists of three key steps: statistically planning and conducting the experiments, developing the suitable mathematical models, and assessing the appropriateness of the developed models [47,48,62,71,72].

(6) $$R=(f\left(x_{1\;}{,x}_{2,\;}x_{3}{,\;x}_{4}\ldots x_{n}\right)\;$$

Here, R represents the responses and *x_i_* represents the input variables under consideration. For each input factor, the low, middle, and high values were expressed by the coded numbers 1, 0, and +1, respectively. It is significant to ensure an accurate estimation of the appropriate functional correlation between the input/independent factors and the predefined responses in order to optimize the system [71,72].

After the experimental runs were completed, the responses were used to develop qualitative models that could be used to correspond the responses of removal percentages (*R*_1_) with fixed carbon (*R*_2_) and yield (*R*_3_) using a second-degree mathematical model, as expressed by Equation (7) [72]:

(7) $$R=b_{0\;}+\sum_{i=0}^{n}b_{1}x_{1}+\sum_{i=1}^{n}b_{ii}x_{1}^{2}+\sum_{i=0}^{n}\times \sum_{j>1}^{n}b_{ij}x_{i}x_{j\;}$$

*R* stands for the anticipated responses, *b*_0_ designates the constant coefficient, *b*_1_ for the linear coefficients, *b_ij_* for the interaction coefficients, *b_ii_* for the quadratic coefficients, and *x_i_*, *x_j_* for the adsorption in coded terms [66]. The proposed number of trials at the centre point for three factors is 5, and the overall number of experiments (N) necessary is 17 (Table 2). The coded and actual levels of three factors, as well as the responses acquired for the BBD experimental design matrix, are presented in Table 2.

#### 3.2.3. Analytical Methods and Physio-Chemical Characterization

To determine the surface characteristics of the produced samples, different analytical methods were used. A field emission scanning electron microscope (FE-SEM) connected with EDX analysis was used to examine the surface morphological variations of final derivatives (SUPRA Zeiss 35-VP; Zeiss Group, Berlin, Germany).

A BET Surface Area Analyser (TriStar II; Micrometrics, Berlin, Germany) was used to determine the BET surface area, micropore, mesopore area, volume, and porosity of the samples. The samples were outgassed under vacuum at 400 °C for 6 h before N_2_ gas adsorption to eliminate moisture content. The Brunauer–Emmett–Teller (BET) method was employed to estimate the surface area and diameter of the pores, while the t-plot approach was used to quantify micropore volume. Iodine number and bulk density, along with P_ZPC_ of the synthesized AKTWAC, were measured [52]. X-ray diffraction (XRD, Bruker AXS-D8 Advance; Bruker Corporation, Kuala Lumpur, Malaysia) operating at 40 kV and 40 mA with a Cu-Kα radiation source was used. XRD analysis was carried out to examine the crystalline phase of the samples. To assess the thermal stability of the materials, thermo-gravimetric analysis (Star Mettler Toledo, Mettler-Toledo (M) Sdn Bhd, Selangor, Malaysia) was carried out. In the TGA study, 5 mg of each sample was heated at 1000 °C, with a heating rate of approximately 5 °C/min under N_2_ gas flow. The fixed carbon (*R*_2_) content, moisture, volatile materials, and ash content were determined using TGA analysis. The percentages of elemental carbon (C), oxygen (O), hydrogen (H), nitrogen (N), and sulphur (S) in AKTW, AKTWC, and AKTWAC, were calculated using an ultimate analysis (Perkin-Elmer; Series II: 2600; Malvern Panalytical, Tokio, Japan). Yield percentage (%) is a physical parameter, and after each experimental run based on Table 2 under different conditions, it was calculated using Equation (8).

(8) $$Yield\;\%=\frac{Initial\;Weight\;of\;the\;Char-Final\;Microwave\;Assisted\;Activated\;Carbon\;Weight}{Initial\;weight\;of\;the\;Char}\times 100$$

**Figure 9:**
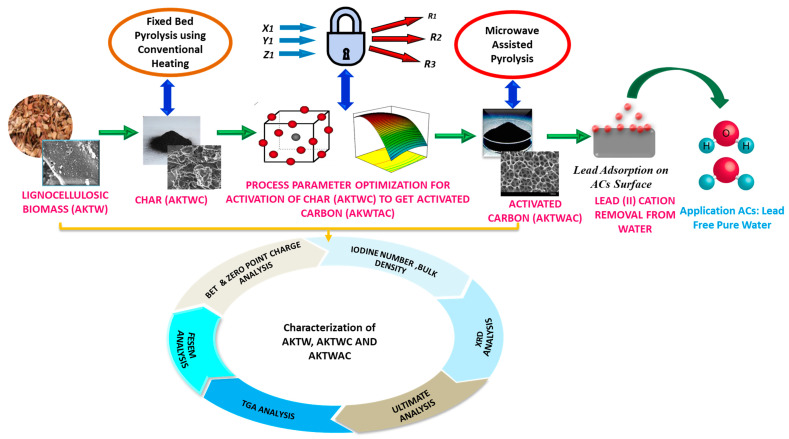
Simplified flow sheet for experimental steps.

All the experiments were triplicated, and average results are tabulated in Table 2. The overall experimental steps are shown in Figure 9.

## 4. Conclusions

The use of microwave heating during the pyrolysis process (MWP) shortened the production time while also generating activated carbon by expanding inaccessible pores and forming new ones. This is due to the bulk, volumetric, internal heating approach induced by the microwave-assisted pyrolysis (MWP) process. The findings indicate a unique technique for producing activated carbon (AC) which is cost-effective and energy-efficient.

The removal percentages of Pb (II) cations (*R*_1_), fixed carbon content percentages (*R*_2_), and yield percentages (*R*_3_) are all controlled by input variables such as power (*x*_1_), radiation time (*y*_1_), and activating agent (K_2_CO_3_) ratio (*z*_1_). Consequently, the level of input variables and the targeted output responses should be assessed initially before designing the AC manufacturing process.

The extent to which the cross-linking and elimination processes occur during the carbonisation and activation process controls the porosity and surface area of the activated carbon (Ass). The results indicate that the optimum process condition leads to the formation of new cavities due to multiple chemical interactions between the AKTWC and K_2_CO_3_, which makes it an efficient adsorbent for elimination of Pb (II) cations from waste effluents. Th Box–Behnken design (BBD) methodology applied in this research illustrates a reasonable and competent strategy to produce activated carbon from *Adansonia kilima (Baobab)* wood chips (AKTW) for removing Pb (II) cations from wastewater using a reduced number of experiments. AKTW has the potential to be used as a feedstock for the production of superior quality ACs, having an enlarged surface area and porous texture.

## Figures and Tables

**Table 1:** Input/independent variables for microwave-assisted pyrolysis (MWP) of AKTWC char, including coded and actual level.

Input/Independent Variables	Code	Unit	Variable Levels (Coded and Actual)			Desired Output/Dependent Variables/Responses		
			−1	0	+1	Removal Percentages (%)	Active Carbon Content (%)	Yield Percentages (%)
Microwave Power	*x*_1_	Watt	550	650	750	*R*_1_	*R*_2_	*R*_3_
Radiation/Residence Time	*y*_1_	Min.	10	12.5	15			
Ratio	*z*_1_	-	1	1.5	2			

**Table 2:** Box–Behnken design (BBD) and experimental responses for microwave-assisted pyrolysis (MWP) of AKTWC char to obtain AKTWAC, including coded and actual Level.

Sample ID	Run	Type of Point	Level (Coded Factors)			Input/Independent Variables (Actual Factors)			Removal Percentage (%)	Fixed Carbon Content (%)	Yield Percentages (%)
						Power *x*_1_, (Watt)	Radiation Time, *y*_1_, (Min.)	Ratio *z*_3_	*R*_1_ (%)	*R*_2_ (%)	*R*_3_ (%)
S-1	1	Center	0	0	0	650	12.50	1.50	85.99	78.77	83.44
S-2	2	Center	0	0	0	650	12.50	1.50	85.98	79.99	84.54
S-3	3	Center	0	0	0	650	12.50	1.50	85.59	78.87	83.33
S-4	4	Center	0	0	0	650	12.50	1.50	85.98	78.32	83.54
S-5	5	Center	0	0	0	650	12.50	1.50	85.77	79.33	82.99
S-6	6	IBFact	0	−1	0	550	10.00	1.50	87.04	80.22	85.88
S-7	7	IBFact	+1	−1	0	750	10.00	1.50	80.99	62.77	80.08
S-8	8	IBFact	+1	0	−1	750	12.50	1.00	84.33	62.76	78.99
S-9	9	IBFact	0	−1	−1	650	10.00	1.00	86.39	76.33	84.06
S-10	10	IBFact	0	+1	+1	650	15.00	2.00	72.33	70.44	76.87
S-11	11	IBFact	−1	0	−1	550	12.50	1.00	86.03	75.66	86.76
S-12	12	IBFact	−1	0	+1	550	12.50	2.00	81.22	70.43	81.09
S-13	13	IBFact	−1	0	0	550	15.00	1.50	82.09	68.44	83.99
S-14	14	IBFact	0	+1	−1	650	15.00	1.00	73.56	70.75	82.45
S-15	15	IBFact	+1	+1	−1	750	15.00	1.50	69.99	61.79	78.89
S-16	16	IBFact	0	0	+1	650	10.00	2.00	74.22	69.87	79.77
S-17	17	IBFact	+1	0	+1	750	12.50	2.00	68.55	61.99	73.32

**Table 3:** Statistical tools for model validation.

Statistical Tools	Output Variables/Responses		
	Removal Percentages	Fixed Carbon Content	Percentage Yield
	*R*_1_	*R*_2_	*R*_3_
Standard Deviation, SD%	1.11	1.20	0.81
Mean	80.94	72.16	81.76
Correlation Coefficient, R^2^	0.98	0.98	0.97
Adjusted R^2^	0.97	0.96	0.94
Coefficient of Variation, CV	1.37	1.67	0.99
Adequate Precision	21.76	18.96	20.26

**Table 4:** Influence of input/independent variables on removal percentages (*R*_1_): analysis of variance (ANOVA) test.

Source	Sum of Squares	Degree of Freedom	Mean Square	F Value	Prob > F	Comments
Model	665.58	9	73.95	60.45	<0.0001	*Significant*
*x*_1_	132.19	1	132.19	108.05	<0.0001	
*y*_1_	117.58	1	117.58	96.11	<0.0001	
*z*_1_	144.42	1	144.42	118.01	0.0003	
*x*_1_^2^	6.20	1	6.20	5.07	0.0591	
*y*_1_^2^	89.91	1	89.91	73.49	<0.0001	
*z*_1_^2^	89.72	1	89.72	73.33	<0.0001	
*x*_1_ *y*_1_	9.15	1	9.15	7.48	0.0291	
*x*_1_ *z*_1_	30.09	1	30.09	24.59	0.0016	
*y*_1_ *z*_1_	29.92	1	29.92	24.46	0.0017	
Residuals	8.56	7	1.22			
Lack of Fit	8.44	3	2.81	88.80	0.0004	
Pure Error	0.137	0.0049				

**Table 5:** Influence of input/independent variables on fixed carbon content percentages (*R*_2_): analysis of variance (ANOVA) test.

Source	Sum of Squares	Degree of Freedom	Mean Square	F Value	Prob > F	Comments
Model	738.36	9	82.04	56.64	<0.0001	*Significant*
*x*_1_	258.10	1	258.10	178.18	<0.0001	
*y*_1_	39.47	1	39.47	27.25	0.0012	
*z*_1_	20.38	1	20.38	14.07	0.0072	
*x*_1_^2^	33.33	1	33.33	168.08	<0.0001	
*y*_1_^2^	46.06	1	46.06	31.78	0.0008	
*z*_1_^2^	64.10	1	64.10	44.25	0.0003	
*x*_1_ *y*_1_	29.16	1	29.16	1.71	0.1063	
*x*_1_ *z*_1_	4.97	1	4.97	13.08	0.0378	
*y*_1_ *z*_1_	9.46	1	9.46	3.43	0.0028	
Residuals	10.14	7	1.45			
Lack of Fit	8.53	3	2.84	14.67	0.0126	
Pure Error	1.61	4	0.40			

**Table 6:** Influence of input/independent variables on yield percentages (*R*_3_): analysis of variance (ANOVA) test.

Source	Sum of Squares	Degree of Freedom	Mean Square	F Value	Prob > F	Comments
Model	184.13	9	20.46	31.34	<0.0001	*Significant*
*x*_1_	87.38	1	87.38	133.86	<0.0001	
*y*_1_	7.20	1	7.20	11.03	0.0127	
*z*_1_	56.23	1	56.23	86.14	<0.0001	
*x*_1_^2^	4.67	1	4.67	7.15	0.0318	
*y*_1_^2^	0.39	1	0.39	0.60	0.4636	
*z*_1_^2^	25.80	1	25.80	39.52	0.0004	
*x*_1_ *y*_1_	0.10	1	0.10	0.19	0.6774	
*x*_1_ *z*_1_	0.30	1	0.30	0.77	0.2987	
*y*_1_ *z*_1_	0.42	1	0.42	0.64	0.4509	
Residuals	4.57	7	0.65			
Lack of Fit	3.22	3	1.07	1.10	0.1471	
Pure Error	1.35	4	0.34			

**Table 7:** Optimization for the microwave-assisted pyrolysis (MWP) process.

Power	Radiation Time	Ratio	Percentage Removal (*R*_1_)			Fixed Carbon Content (*R*_2_)			Yield Percentages (*R*_3_)		
(Watt)	(Min.)	(-)	Predicted	Experimental	Error	Predicted	Experimental	Error	Predicted	Experimental	Error
568.59	10.13	1.32	87.73	85.66	2.35	80.98	80.02	1.18	86.85	85.90	1.09

**Table 8:** Surface textural properties analysis.

Sample	S_BET_ (m^2^/g)	S_mic_ (m^2^/g)	Langmuir Surface Area (m^2^/g)	External Surface Area (m^2^/g)	S_mic_/S_BET_ (%)	V_mic_ (cm^3^/g)	V_tot_ (cm^3^/g)	Diameter (nm)	Bulk Density (g/mL)	Iodine Number (mg/g)	P_ZPC_
AKTWC	448.79	209.99	699.89	238.81	46.79	0.07	0.16	4.76	-	-	-
AKTWAC	1390.76	798.67	1882.91	592.09	55.26	0.49	0.96	6.73	0.07	1297.87	4.6

**Table 9:** Thermo-gravimetric (proximate analysis—TGA) of AKTW, AKTWC, and AKTWAC.

Proximate Analysis (TGA)	AKTW	AKTWC	AKTWAC
Carbon Content (%)	51.92	70.70	80.02
Moisture (%)	3.88	2.87	1.76
Volatile Matter (%)	36.98	14.55	2.07
Ash Residues (%)	7.22	11.88	16.21
*dtg_max_*	347.38	371.22	378.87
*Elemental Analysis*			
*C*	53.98	68.78	81.03
*H*	6.87	4.89	1.12
*N*	2.12	0.78	0.24
*O*	35.05	24.52	17.29
*S*	1.98	1.03	0.32

## Data Availability

The data presented in this study are available on request from the corresponding author. The data are not publicly available due to privacy.
